# The Future of Insurance Practice

**Published:** 1916-09-02

**Authors:** John MacKeith

**Affiliations:** 15 Cavendish Place, Cavendish Square, W.


### THE EDITOR'S LETTER-BOX.

### The Future of Insurance Practice.

*To the Editor of* The Hospital.

Sir,—I enclose copy of a letter I have to-day sent to the National Health Insurance Commission (England), and will be much obliged if you can find room for it in The Hospital.—I am, yours truly, John MacKeith.

15 Cavendish Place, Cavendish Square, W.,
August 29, 1916.

